# Maternal SSRI use during pregnancy and offspring depression or anxiety disorders: A review of the literature and description of a study protocol for a register-based cohort study

**DOI:** 10.1016/j.reprotox.2023.108365

**Published:** 2023-03-22

**Authors:** Subina Upadhyaya, Alan Brown, Keely Cheslack-Postava, Mika Gissler, David Gyllenberg, Emmi Heinonen, Joonas Laitinen, Ian McKeague, Susanna Hinkka-Yli-Salomäki, Andre Sourander, Aleksi Tornio, Heli Malm

**Affiliations:** a Research Centre for Child Psychiatry, Department of Child Psychiatry, University of Turku, INVEST Flagship Centre, Turku, Finland; b Department of Psychiatry, New York State Psychiatric Institute, Columbia University Irving Medical Center, New York, NY, USA; c Department of Epidemiology, Columbia University, Mailman School of Public Health, New York, NY, USA; d Finnish Institute for Health and Welfare, Helsinki, Finland; e Columbia University Mailman School of Public Health, Department of Biostatistics, New York, NY, USA; f Institute of Biomedicine, University of Turku and Unit of Clinical Pharmacology, Turku University Hospital, Turku, Finland; g Teratology Information, Helsinki University and Helsinki University Hospital, Department of Emergency Medicine Services, Helsinki, Finland; h Department of Clinical Pharmacology, Helsinki University and Helsinki University Hospital, Helsinki, Finland; i Individualized Drug Therapy Research Program, Faculty of Medicine, University of Helsinki, Helsinki, Finland

**Keywords:** SSRI, Maternal, Pregnancy, Offspring, Depression, Anxiety

## Abstract

Previous studies examining the relationship between in utero exposure to selective serotonin reuptake inhibitors (SSRI) and long-term offspring depressive or anxiety behaviors are inconclusive. We aimed to critically review the findings of previous studies and describe a new study protocol to investigate the association of prenatal SSRI exposure and offspring depression or anxiety using data from several Finnish national registers. The study includes 1,266,473 mothers and their live-born singleton offspring, born in 1996–2018. The study cohorts include the prenatally SSRI exposed group and three comparison groups: 1) depression exposed/antidepressants unexposed, 2) unexposed to antidepressants or antipsychotics and depression, and 3) discordant siblings. We aim to examine whether depression in prenatally SSRI exposed children is more common or severe than depression in the offspring of mothers with depression but without SSRI exposure. We aim to disambiguate the effects of maternal SSRI from the effects of maternal depression, severity of maternal depression and familial loading history of psychiatric disorders by including data from first-degree relatives of prenatally SSRI exposed and unexposed children. Associations between exposure and outcome are assessed by statistical modeling, accounting for within-family correlation. The study has potential public health significance and in guiding clinicians in considering treatment options for pregnant women.

## 1. Introduction

Antidepressants have been prescribed increasingly during the past few decades for treatment of moderate to severe perinatal depression [1]. Approximately 7–13% mothers experience depression during pregnancy [2] and 6–10% of all pregnant women are prescribed antidepressants [3–5]. The most commonly used antidepressants are selective serotonin reuptake inhibitors (SSRIs). SSRIs are generally considered safe during pregnancy with no clearly established teratogenic effects. However, research results related to long term neurodevelopment have been conflicting. SSRIs pass to the fetus resulting in concentrations close or similar to that of the mother’s and may perturb serotonin (5-HT) signaling in the developing brain, possibly increasing the risk of depression-like behavior [6]. Further, infants exposed prenatally to SSRIs are at an increased risk of needing treatment in neonatal care, and some of the adverse neonatal effects associated with maternal SSRI use may act as moderators for later neurodevelopment [7].

As we detail below, there is inconsistency in the previous literature regarding antidepressant use during pregnancy, and offspring depressive or anxiety behaviors may be related to methodological considerations. Here we critically review previous studies and describe a study protocol investigating the association of prenatal SSRI exposure and offspring depression or anxiety using national register data from Finland.

## 2. Literature search and findings from previous studies

We searched published literature (PubMed) on antidepressants or SSRI use during pregnancy, including original studies and focusing on long-term depression, depression-like behavior, anxiety disorders and emotional problems. The literature search was performed in PubMed until 7.10. 2021, using the following search terms: (((SSRI OR antidepressant* OR antidepressive medication) AND (pregnancy OR antenatal OR pregnant OR prenatal)) AND ((offspring OR child) AND (depression OR depress* OR psychiatric OR internalizing OR anxiety OR psychosocial))).

We included human observational studies that used longitudinal study designs and were published in peer-reviewed journals. The articles were written in English and examined associations between maternal antidepressants or SSRI use during pregnancy and offspring long-term depression, depression-like behavior, anxiety disorders and emotional problems. We excluded any cross-sectional studies, case reports, conference abstracts, editorials, comments, letters and reviews with no relevant primary data. All studies were screened for full-text assessments based on inclusion and exclusion criteria by one reviewer (S.U) and a senior researcher (H.M) was consulted in cases of uncertainty. The relevant data were extracted and summarized based on author, year, country, data source, study period, study design, sample size, follow up age range, exposure group, reference group, primary outcomes, covariates, and results (Table 1).

The electronic search in PubMed yielded 919 articles. After full text screening, we identified and reviewed a total of 15 studies examining the association between maternal SSRI or other antidepressant exposure during pregnancy and offspring depressive, anxiety disorders or behavioral disorders, summarized in Table 1. Three register-based studies examined the association between prenatal exposure to SSRI and offspring mood, depression, or anxiety disorders [8–10]. The Finnish register-based study included 15,729 children exposed to SSRI prenatally [9]. The results showed an association between prenatal SSRI exposure and increased offspring depression until 14 years of age when compared to those unexposed to SSRIs but exposed to maternal depression, and to those whose mothers had discontinued SSRI before pregnancy. The association was not statistically significant for offspring anxiety disorders (Table 1). While these comparison groups were chosen to control for maternal underlying illness, register-based studies only allow adjustment for proxies of maternal illness severity. Two Danish register-based studies with partially overlapping material included nearly one million children, of whom more than 15,000 were exposed to SSRIs through maternal use during pregnancy [8]. Both compared children of mothers with continuous use of antidepressant during pregnancy to those whose mothers discontinued antidepressants prior to pregnancy, and found a significant association between prenatal antidepressant exposure and offspring mood disorders or affective disorders. Both studies also examined the association by timing and duration of antidepressant exposure and overall psychiatric disorders or affective disorders in children until 18 years of age. The results, however, were not specific to depression or depressive disorders. Of 12 other small studies based on cohort design, four studies reported significant associations between exposure to SSRI or other antidepressants and offspring internalizing or emotional problems [11–14], while others reported no association [15–22]. The overall sample size and the individuals exposed to SSRIs or other antidepressants in these studies were small. They assessed offspring’s internalizing behaviors or emotional problems and were categorized heterogeneously, therefore it is difficult to make comparisons. These studies used parental rated report for child’s behavior problems, and maternal self-report for depression.

A recent review [23] concluded that even though the evidence from rodent studies suggests that prenatal exposure to antidepressants may increase depressive behavior in the offspring, the evidence from human studies shows controversial findings. This is the case especially when maternal mood is controlled for [23]. Furthermore, in many studies, statistical power has been limited due to small sample sizes, and sex differences in vulnerability to depression following maternal SSRI treatment has not been explored. Moreover, previous studies pose challenges related to attrition bias, recall bias, short follow up time and outcome reporting bias, including maternal report of offspring behavioral problems and maternal self-report for depression or psychiatric illness. Further, the inability to account for severity of maternal depression may seriously bias the results. Most of the previous studies are also based on relatively young populations. Depression generally starts to emerge at puberty and early adolescence and there is a need to extend follow-up until adulthood.

## 3. Present study

### 3.1. Aims of the present study

The main aim of the study described here is to examine if the previously reported increased risk of depression among offspring exposed to maternal SSRI’s [9] continues to rise from age 14 onwards and whether depression in prenatally exposed children is more severe than depression in the offspring of mothers with depression but no SSRI medication. We aim to disambiguate the effects of maternal SSRI from the effects of maternal depression, severity of maternal depression and familial loading history of psychiatric disorders by using information from several nationwide registers, including first-degree relatives of prenatally SSRI exposed children (fathers, siblings). We examine the role of drug-related properties and offspring sex as potential contributors to the vulnerability. We also investigate the most susceptible time window of exposure for study outcomes. Further, we evaluate the role of pregnancy and neonatal complications associated with SSRI use during pregnancy as mediating factors of maternal SSRI exposure and offspring psychiatric outcomes.

### 3.2. Methods

**Fig. 1.**
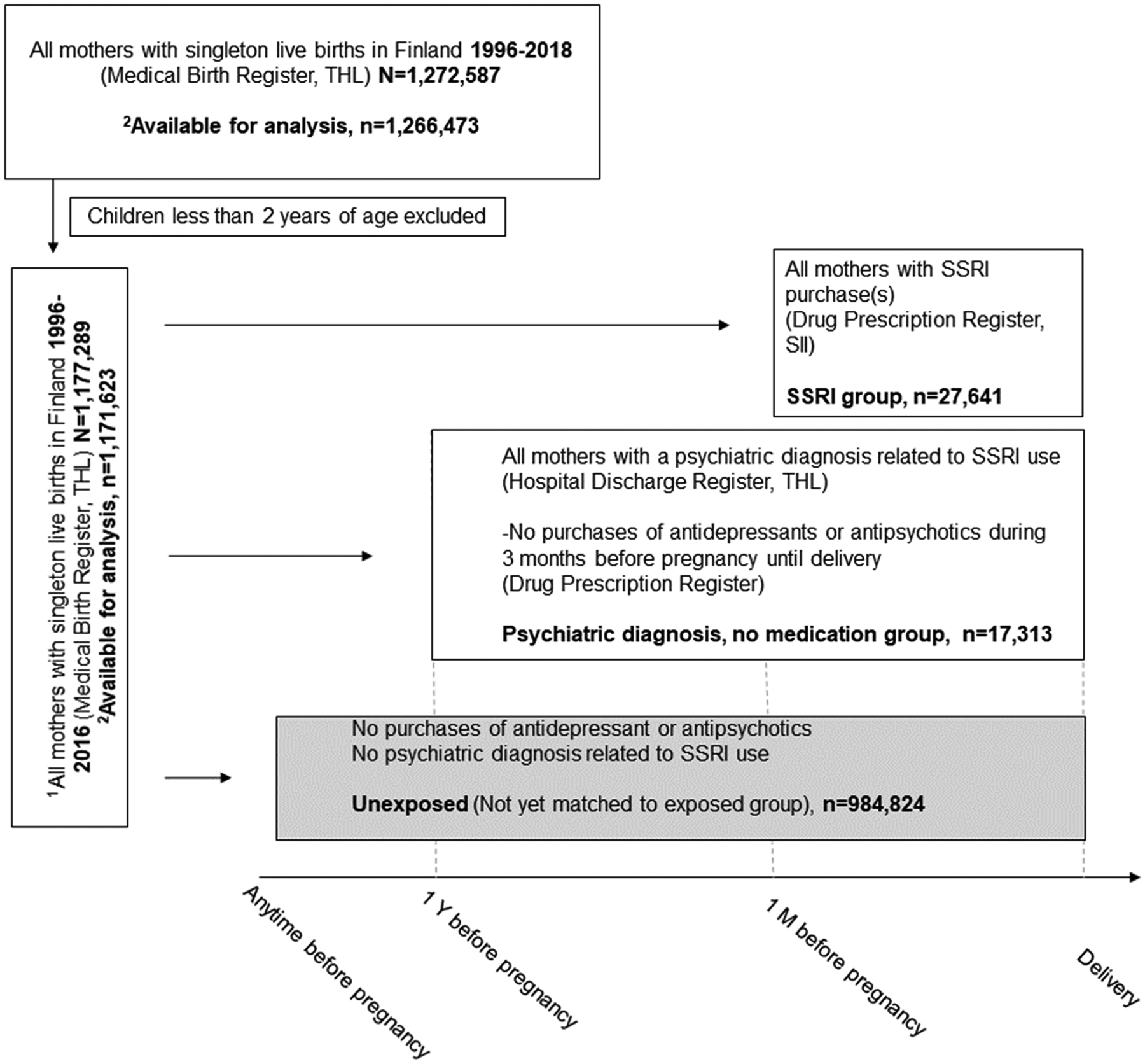
Flow chart of the register-based information and the time-window criteria for definition of exposure groups.

This is a nationwide register-based cohort study. In Finland, all births in the country and all psychiatric diagnoses and psychiatric drug prescriptions among the cohort are registered in national, population-based registers, which forms the basis of this investigation. The total sampling frame includes all singleton, live-born infants born in 1996–2018. Because diagnosis of depression or anxiety is unlikely before 2 years age, we included only those born in 2016 or earlier to allow follow up to at least up to 2 years age (Fig. 1). The study has been registered in the EU PAS Register (EUPAS33119). Fig. 1 illustrates in detail the study design, definition of exposure groups and register-based information.

#### 3.2.1. Description of data sources

The national registers used to collect information on study variables are summarized in Table 2. These include:

##### The national Medical Birth Register, MBR

*The national Medical Birth Register, MBR,* maintained by the Finnish Institute for Health and Welfare (THL) is a computerized registry collecting data on maternal demographic characteristics, medical history including reproductive history, health-related behaviors, diagnoses during pregnancy and delivery, and on neonatal outcome data up to age seven days. Data in the MBR are collected in a standard form from all maternity hospitals and include all hospital births and the occasional homebirths. Practically all live births and stillbirths with gestational age of ≥ 22 weeks or birth weight of ≥ 500 g are included in the register. The definitions and variables included in this registry are based on established international concepts and use the 10th version of the WHO International Statistical Classification of Diseases and Health Problems (ICD). Extensive review of the data, including cross-checking with the data from the Finnish Population Register and Cause-of-Death Register at Statistics Finland, indicate that the data are virtually complete [24, 25]. Data for mothers and infants are collected through years 1996–2018 for all singleton live births (N = 1266,473).

##### The national Hospital Discharge Register, HDR (THL)

*The national Hospital Discharge Register, HDR (THL)* contains a hospital identification code, data on admission and discharge dates, and primary and secondary diagnoses at discharge. The register covers all hospital inpatient episodes in public and private institutions and outpatient hospital visits in public hospitals. The diagnoses are coded using ICD-8 (1969–1986), ICD-9 (1987–1995), and ICD-10 since 1996. Data on hospital discharges are available since 1967, and data on all contacts in outpatient clinics are available since 1998. These registries are used to identify the recorded diagnoses for all psychiatric hospital admissions and outpatient psychiatric contacts for mothers, fathers and all children, and somatic diagnoses for mothers and children. The HDR data have been validated for psychiatric diagnoses and are considered good [26–28]. All psychiatric diagnoses of mothers, fathers and children are also collected from the Primary Health Care Register, available from 2011 onwards. Further, diagnoses for somatic chronic diseases are collected for the mothers.

##### The national Drug Reimbursement Register, DRR

*The national Drug Reimbursement Register*, *DRR,* maintained by the Social Insurance Institution in Finland (KELA) contains data on 99% of reimbursed prescription drug purchases (Finnish statistics on medicines 2020). Prescription-only medicines deemed necessary for the treatment of an illness are reimbursed under the Social Insurance Scheme that covers all permanent residents in Finland. Drug purchases are reimbursed concomitantly upon purchase at pharmacy and drugs are supplied to the patient for a maximum of three months at a time. The data in the register include the International Anatomic-Therapeutic-Chemical (ATC) classification code indicating the generic name of the drug, the dose prescribed, and information on possible special reimbursement status including indication for treatment. Over-the-counter drugs or medications given to institutionalized persons are not included in the register. Data for purchases of antidepressants, antipsychotics, anxiolytics, hypnotics and sedatives, and antiepileptic drugs are collected for mothers, fathers and children through years 1994–2018, as are purchases of known or suspected teratogens for mothers. Since 1964, KELA also maintains *the Special Reimbursement Register* with data on several chronic illnesses requiring continuous drug treatment. Special reimbursement is granted to persons with a diagnosed chronic disease, such as diabetes or epilepsy, among others. Data from this register are collected for mothers, fathers, and children for years 1964–2018. Other registers included in the study are the Register for Congenital Malformations, the Population Information System and Statistics Finland (Table 2).

#### 3.2.2. Data linkages and management

Data related to pregnancy and infancy are obtained from the Medical Birth Register. Data on SSRI purchases are collected from the Drug Reimbursement Register. The follow-up for psychiatric disorders is based on collection of these diagnoses from the Hospital Discharge Register and from the Register of Primary Health Care Visits. Data on major congenital anomalies are collected from the Register of Congenital Malformations, and parental background data from the other registers. All data are linked by a unique personal identification number, which is assigned to all citizens and permanent residents of Finland. The data linkages and preliminary data processing are being performed by the Finnish Social and Health Data Permit Authority (Findata). The data are pseudonymized and are securely stored and managed by Findata. The pseudonymized research data are available in the Findata secure operating environment by remote access.

Findata issues permits for the secondary use of social and health data, and the data combined from Finnish registers that is subject to a permit in a secure manner and pre-processes these ensuring the privacy of citizens. A permit can be issued for the register-based studies in accordance with the Act on secondary use of health and social data (552/2019).

#### 3.2.3. Ethical considerations

The study protocol has been approved by the Ethics Committee of the Turku University Hospital (23/1801/2020) and the Institutional Review Board of the New York State Psychiatric Institute.

#### 3.2.4. Exposure and comparison groups

The study includes the prenatally SSRI exposed group and three comparison groups: 1) Depression exposed/antidepressant unexposed, 2) unexposed to antidepressants or antipsychotics and depression, and 3) discordant siblings. These study groups are described in detail below. In addition to these comparison groups, we examine a separate category of children of mothers exposed to SSRI prior to pregnancy, but not during pregnancy. This group includes mothers who used antidepressants (ATC codes N06A, N06CA) prior to pregnancy (during the period of one year to three months before pregnancy) but not during pregnancy. We also examine the association of paternal SSRI use as a negative comparison group for fetal drug exposure.

##### SSRI-exposed group

Mothers in this group have prescriptions purchased for one or more purchases of SSRIs (Anatomical Therapeutic Chemical (ATC) code N06AB; including fluoxetine, N06AB03; citalopram, N06AB04; paroxetine, N06AB05; sertraline N06AB06; fluvoxamine N06AB08; escitalopram N08AB10) during the period from 30 days before pregnancy until the end of pregnancy. This group includes 27,641 subjects.

##### Comparison Group 1 (Depression exposed/antidepressants unexposed)

Comparison Group 1 (Depression exposed/antidepressants unexposed) includes subjects (n = 17,313) exposed to maternal depression [diagnosis of depression or psychiatric disorder related to depression, obtained from the HDR (ICD-10: F20–48; ICD-9: 295, 296, 297, 2988A, 2989X, 3000, 3001, 3002, 3003, 3004A, 3006, 3007, 3008, 3009, 3013C, 3078A, 3090A, 3092C, 3092D, 3092E, 3098A, 3098X, 3099X; ICD-8: 295, 296, 297, 29800, 29820, 29830, 29899, 299, 300, 305, 30799, 79020) from one year before pregnancy until discharge (≤3 weeks) from hospital after delivery], but not exposed to SSRIs [no purchases of any antidepressants (ATC codes N06A, N06CA) or antipsychotics (N05A) from three months prior to pregnancy until the end of pregnancy] during pregnancy.

##### Comparison Group 2 (Unexposed)

Comparison Group 2 (Unexposed) includes subjects (n = 984,824) unexposed to antidepressants and antipsychotics and unexposed also to depression or psychiatric disorders related to depression at any time prior to or during pregnancy. Ten unexposed are matched with one SSRI exposed by sex and date of birth (+/− 30 days).

##### Comparison group 3 (Discordant siblings)

This group includes children of mothers who purchased SSRIs during 30 days before pregnancy until birth in another pregnancy (SSRI exposed), but not in this pregnancy, i.e., in this pregnancy the mother has no purchases of antidepressants during 30 days before pregnancy until birth. This group includes 22,409 subjects.

#### 3.2.5. Outcomes

##### Depressive disorders.

We utilize registry diagnoses [depressive disorder (ICD-10 F32), and other depressive disorders (F33-F39)] for the diagnosis of depression. **Anxiety disorders** are based on ICD-10 diagnoses F40 (phobic anxiety disorders), F41 (panic disorder, generalized anxiety disorder, other mixed/unspecified anxiety disorders), as received from the registers.

#### 3.2.6. Covariates

Extensive data on covariates on all pregnancies and on mothers, fathers, and siblings before, during, and after pregnancy are obtained from the national registers. The study covariates and their categorization are presented in Table 3. Demographic and reproductive characteristics include maternal age/paternal age at offspring birth, information of previous pregnancies, mothers’ somatic illnesses, parental socioeconomic status (education, place of residence), marital status of parents, maternal municipality of residence, previous births, maternal smoking, and perinatal and neonatal complications. Covariates related to medications include maternal antidepressant use after pregnancy and exposure to other known or suspected teratogenic medications during pregnancy, including antiepileptic drugs. Data on parental psychiatric history and substance abuse are included.

#### 3.2.7. Assessing maternal illness severity

Illness severity proxies include age of onset (first diagnosis) of maternal depression, specialized service use for depression-related disorders and specific diagnoses occurring one year before pregnancy until delivery (hierarchical, mutually exclusive variable including psychoses, affective disorders, and anxiety and neurotic disorders). Further, information about hospitalization, intentional self-harm requiring hospital treatment, and treatment resistance defined by the number of different antidepressants or adjunctive mood stabilizers are used to adjust for maternal illness severity.

#### 3.2.8. Statistical analyses plan

We will produce a Kaplan-Meier plot of the cumulative incidence of the outcome by exposure group to visually compare the risk of depression between exposure groups. Cox regression is used for each outcome to examine the association between exposure group and time-to-event. To account for within-family correlation, children of the same mother are treated as clusters. Robust sandwich estimates of the variance of the estimated Cox regression parameters are used for this purpose. Hazard ratios (HRs) and 95% confidence intervals (CI) for the association between exposure and outcomes are calculated. A p value of < 0.05 is considered statistically significant.

The associations between maternal exposure categories and offspring depressive disorders or anxiety disorders are examined by using linear and logistic regression, respectively, adjusting for covariates as described above. The covariates shown in Table 3 are each examined and are selected to be included in the model if they are associated with both exposure and outcomes (p < 0.1). Additional data are used to disambiguate from maternal SSRI use the contributions of maternal illness severity, and familial loading of psychiatric disorders on offspring psychiatric outcomes. We address gestational timing of SSRI exposure, serotonin selectivity of the antidepressant and level of affinity of the antidepressant to the serotonin transporter, dose and duration of SSRI exposure and offspring sex to determine other possible factors affecting the vulnerability of offspring to psychiatric outcomes. We also examine pregnancy and neonatal complications including preterm birth, Cesarean section, low Apgar score (0–6 at 5 min), neonatal breathing problems, and monitoring in a neonatal intensive care unit, as appropriate, as putative mediators for any observed relationships between maternal SSRI exposure on risk of offspring psychiatric outcomes.

All analyses are being conducted in SAS (SAS 9.4, SAS Institute, Cary, NC, USA).

#### 3.2.9. Statistical power

There are 27,641 subjects in the SSRI exposed group and 17,313 subjects in the group exposed to depression but not to antidepressants. The study has 90% power to detect 1.18-fold increase in hazard of depressive disorders, that have prevalence of 3.76%, and 99.9% power to detect 1.28-fold increase in hazard of anxiety disorders, that have prevalence of 4.26% in the study cohort (alpha=0.05, two-sided).

### 3.3. Discussion

To our knowledge, no previous studies have examined associations between maternal SSRI use and offspring depression with follow-up extending into young adulthood, with the possibility of controlling for confounding related to several maternal, paternal or neonatal factors, familial loading, and a large population base allowing to the examination of timing of exposure and pharmacological characteristics. The present study utilizes a large, national birth cohort with study subjects followed from the prenatal period up to a maximum of 22 years of age.

#### 3.3.1. Large sample size and statistical power

Most of the previous studies were based on small sample sizes, and thus statistical power was limited for subgroup analyses. Moreover, sex differences in vulnerability to offspring psychiatric outcomes following maternal exposure to SSRIs or antidepressants were generally not explored. Statistical power was also not fully adequate to evaluate relationships between maternal SSRI use and other offspring psychiatric outcomes, including anxiety disorders. The high dropout rate or low response rate reported in several previous studies, limiting the sample size at the end of the follow up period, is another important methodological limitation of several studies. The previously published register-based studies were not large enough to assess the timing of exposure [9] or did not specifically address offspring depression or anxiety disorders [8,10].

The main strength of the present study includes a large and population-based cohort that provides ample opportunity for sex specific analyses, stratified analyses, and using several comparison groups, including a comparison group of depressed mothers not taking antidepressants during pregnancy. Furthermore, all the diagnosed cases will be ascertained by the national registers, thereby the attrition rate is low.

#### 3.3.2. Assessment of study outcomes

Many previous studies relied on clinical, rather than population-based samples and did not investigate national samples with universal coverage of psychiatric disorders and medical illnesses, increasing the likelihood that cases diagnosed with psychiatric disorders could have been missed. Of note, only three previous register-based studies used nationwide register-based diagnoses for defining offspring mood disorders, depression, or anxiety disorders [8–10]. The results from two Danish studies, however, did not separate depression from mood disorders [8,10]. Other clinical studies used the mother-rated child behavioral checklist (CBCL) and strengths and difficulties questionnaire (SDQ) to assess child behavioral outcomes, which is subject to reporter bias [11–22]. Moreover, internalizing behavior, emotional problems or affective problems assessed in these studies were categorized heterogeneously making comparisons difficult. In Finland, data on all psychiatric diagnoses can be ascertained. The large amount of register data, including data both from specialized services and primary health care increases the representativeness of the sample together with diminishing selection bias.

#### 3.3.3. Age of follow-up

In previous studies, children were infrequently followed up past childhood and therefore the age of highest risk for depression following maternal SSRI exposure has not been included. The majority of previous studies have carried out the child assessment for internalizing behavior at an early age, from 0 to 6 years (Table 1), reporting conflicting findings regarding offspring outcomes. Most of the studies used maternal report of the CBCL or SDQ in assessing outcomes in 1.5–5-year-old children and showed no differences between children prenatally exposed and unexposed to antidepressant medications [15,16,18,22]. However, some studies reported a higher rate of internalizing symptoms at an early age in children of mothers with antenatal depression regardless of medication, when compared to children of mothers without depression. This is important, as it has been suggested that consequences of early life exposures to SSRIs in the developing brain may not manifest immediately but may occur later during adolescence or even in adulthood [29]. Three studies using national register data have been published with a longer follow-up [8–10]. The longest follow-up period in a previous register-based study was until age 18 years; thus, the vast majority of the age of risk for depression was not covered. The follow-up period of the present study is extended up to 22 years, which also allows us to examine offspring vulnerability to depression diagnoses following maternal SSRI by the age of onset of depression diagnosis.

#### 3.3.4. Timing of exposure and pharmacological factors

It is not known if SSRI medications carry a risk throughout the gestational period, or if there is a critical developmental time window when the fetus might be more vulnerable and there is a paucity of studies differentiating between exposure during early gestation versus late gestation. Only one study from Norway based on the MoBa Birth Cohort data examined the association between mid- or late pregnancy SSRI exposure and offspring outcomes and showed a significantly increased risk of anxious/depressed behaviors (adjusted β 0.50; 95% CI 0.04, 0.95) at the age of 5 years in children with late pregnancy exposure when compared to children who were unexposed. This association was not seen for mid-pregnancy exposure [14]. Since this is the only finding related to timing of the exposure, more evidence of potentially important periods of fetal vulnerability to SSRI exposure is needed. The present study examines the gestational timing and duration of SSRI exposure, in each trimester and subsequent risk of offspring psychiatric outcomes. The large sample size in the present study allows us to examine separately gestational periods of vulnerability to SSRIs, serotonin selectivity and half-life of individual antidepressants, as well as dose and duration of exposure.

#### 3.3.5. Maternal depression severity and other confounding influences

Distinguishing between the effects of maternal mental illness and illness severity and drug effects has been a challenge in previous studies, including the register-based studies [8–10]. A study comparing three groups of mothers exposed to either SSRI or depression (depressed, exposed to SSRI, exposed to venlafaxine) during pregnancy to healthy control mothers reported that children from all three comparison groups exhibited more clinically significant behavioral problem as measured by the CBCL, compared to children of the healthy mothers. That study also found that it was the severity and timing of the depression that affected child behavior rather than dose and duration of antidepressant treatment [19]. It should be noted that the number of participants in each comparison group were rather small (n = 62). Previous studies have mostly relied on information obtained from validated child behavior checklists completed by mothers/ parents. Parent-reported scores however may not reflect actual child behavior. This may be the case especially when the parent experiences anxiety or depression at time of the assessment. Studies controlling for maternal symptoms at the assessment have indicated that mothers who report anxious or depressive symptoms are also more prone to report problematic internalizing behavior in the offspring [17]. There is also a lack of evidence in prior investigations on whether maternal SSRI exposure was related to severity of depression in the offspring.

Previous studies were also challenged by the inability to more effectively disambiguate from maternal SSRI use the contributions of familial loading on offspring psychiatric outcomes. Including data from first degree relatives allows consideration of a large number of potential confounders related to the multifactorial origin of depression. Sibling discordance studies are suitable for examining the relation of prenatal exposures to offspring outcomes. While some features of the family environment differ between siblings, same-sex siblings share enough stable aspects of the family environment as well as genetic predisposition to reduce the potential for confounding [30,31]. Only two previous studies examined the association of maternal SSRI or antidepressants and offspring depression using sibling designs [11,18] (Table 1). Moreover, the present study includes data on paternal antidepressant use during the mother’s pregnancy, further enabling control for confounding by family related factors.

It is important to note that the unique possibility of data linkages between several national registers in the present study will allow us to incorporate several potential mediating factors in the analyses. For example, prior studies have not considered important mediating factors related to neonatal complications. The register-based design allows us to evaluate whether the associations between maternal SSRI exposure and offspring psychiatric outcomes are mediated by neonatal complications due to SSRI toxicity or withdrawal.

#### 3.3.6. Possible limitations of present study

There are also some potential limitations of the study that should be considered. First, we rely on the registry information to ascertain psychiatric outcomes. However, several psychiatric diagnoses have been validated using research-based interviews and chart reviews [27,32,33] and the completeness and accuracy of overall psychiatric outcomes in HDR were shown to be good [34]. Second, information on maternal antidepressant use during pregnancy is obtained from registers and we are not able to verify whether purchased medications were taken by the mothers. To address this limitation, we will conduct additional analyses restricted to mothers with more than one prescription purchase for the same SSRI, which will indicate a greater likelihood of taking the medication. Third, while we aim to control for the severity of maternal depression by including a large number of covariates serving as proxies for depression severity, we cannot fully adjust for depression severity in the register-based setting. Further, as in all cohort studies, residual confounding remains a possibility. We aim to minimize residual confounding by adjusting for several other potential covariates including familial loading for psychiatric disorders, by using sibling analyses, comparisons of offspring outcomes between maternal and paternal antidepressant use, and access to a large number of additional covariates in the database. Fourth, the generalizability of our findings remains somewhat limited, as our sample is drawn from Finland, where the vast majority of the population are Caucasians, with relatively high rates of depression [35].

#### 3.3.7. Implications

The risk of using antidepressant medication during pregnancy must always be weighed individually and against the established risks of under-treated maternal depression. Given the widespread use of SSRIs during pregnancy, it is of utmost importance to confirm also the long-term safety of prenatal SSRI exposure. The results will help expectant mothers and their doctors in balancing the risks and benefits when considering treatment options.

## Figures and Tables

**Table 1:** Summary of reviewed articles.

Author Country Data source Study period Study design	Follow up (age range) Children (n)	Exposure group (n)	Reference group (n)	Primary outcome (s)	Covariates/confounders	Results	Comments
Rommel et al. 2021 Denmark National registers 1998–2016 Cohort	0–18 years 42,988	Any AD (15,892) SSRI (14,818) Paternal AD use	Exposed to AD within 2 years prior to pregnancy (discontinuation group)	Diagnosis of: Mood disorders (F30–F39) Neurotic, stress-related and somatoform disorders (F40–F48) Mixed disorders of conduct and emotions (F92) Emotional disorders with onset specific to childhood (F93) Use of ADs/anxiolytics	Maternal age at delivery, primiparity, in- and outpatient psychiatric treatment from 2 years before pregnancy until delivery, prescriptions for other psychotropic drugs during pregnancy, prescriptions for antiepileptic drugs during pregnancy, number of non-psychiatric hospital visits during pregnancy, smoking during pregnancy, marital status, highest education, calendar year of delivery and paternal psychiatric history at the time of delivery.	Based on diagnosis: -Affective disorder, any AD: (HR 1.20; 95% CI 1.08–1.34) SSRI monotherapy (HR 1.19; 95% CI 1.06–1.34) -Mood disorder, any AD: (HR 1.46; 95% CI 1.05–2.03) -Neurotic, stress-related and somatoform disorders, any AD: (HR 1.18; 95% CI 1.01, 1.39) -Emotional disorder, any AD: (HR 1.57; 95% CI 1.18–2.10) Based on offspring use of antidepressant or anxiolytic: -Affective disorder, any AD:(HR 1.47; 95% CI 1.18–1.83) Paternal use of AD during pregnancy: -Affective disorder: (HR 1.29; 95% CI 1.12–1.49)	Outcome defined by diagnosis or drug use (+) AD exposure and affective disorder by timing, duration and type (+) Study material overlapping with Liu et al. 2017 (−) Results suggest familial/environment-related factors rather than prenatal AD exposure to be important
Liu et al., 2017 Denmark National registers 1998–2012 Cohort	0–16.5 years 905,383	Any AD (21.063) SSRI monotherapy (16,154) Continuing AD during pregnancy (17,560) AD use started during pregnancy (3503) Paternal use of AD	Discontinuing AD before pregnancy (30,079) Unexposed (854,241)	Diagnosis of Mood disorder (F30–F39) Neurotic, stress-related and somatoform disorders (F40–48) Behavioral and emotional disorder (F90–F98)	Maternal age at delivery, primiparity, maternal psychiatric history at delivery, inpatient and outpatient psychiatric treatment from 2 years before pregnancy until delivery, dispensing of other psychotropic prescriptions during pregnancy, dispensing of antiepileptic prescriptions during pregnancy, number of non-psychiatric hospital visits during pregnancy, smoking during pregnancy, place of residence, marital status, highest education, income, calendar year of delivery, and paternal psychiatric history at time of delivery.	Continuing AD compared to discontinuing group: -Mood disorder (HR 2.76; 95% CI 1.59–4.78) -Neurotic, stress-related and somatoform disorders (HR 1.62; 95% CI 1.36–1.94) -Behavioral and emotional disorder (HR1.13; 95% CI 1.01–1.27) Paternal antidepressant use during pregnancy associated with overall psychiatric, somatoform, and behavioral disorders.	AD exposure and psychiatric diagnoses by timing and duration (+) Some subgroup analyses based on quite small numbers of cases (−) Unable to control for confounding due to alcohol use (−) Results suggest a role in shared familial or environmental factors.
Malm et al., 2016 Finland 1996–2010 National registers Cohort	0–14 years 64754	SSRI exposed (15,729)	Psychiatric disorder, no AD use (9651) SSRI discontinued (7980) Unexposed (31,394)	Diagnosis of Depressive disorders and unspecified affective disorders (ICD-10 F32–39) Anxiety (F40–41) Autism spectrum disorder (F84, but excluding Rett syndrome, F84.2) Attention-deficit/hyperactivity disorder (F90)	Sex, socioeconomic status, smoking during pregnancy, maternal history of other psychiatric diagnosis, maternal history of substance abuse, paternal history of psychiatric diagnosis, neonatal complications.	-Depression: compared with the psychiatric disorder, no AD group, adjusted (HR 1.78; 95% CI 1.12–2.82) compared with the SSRI discontinued group (HR 1.84; 95% CI 1.14–2.97) -Anxiety: no increased risk in SSRI exposed compared to psychiatric diagnosis, no AD group or to SSRI discontinued group	Maternal illness considered as a covariate (+) Trimester-based timing of exposure not analyzed (−) No analyses on individual SSRI level (−) No data on postnatal environment (−)
Internalizing behavior/Emotional problems—categorized heterogeneously							
van der Veere et al., 2020 Netherlands Pregnant women recruited to study 2007–2010 (n = 111) Prospective longitudinal cohort	2.5 years 102	SSRI exposed (61)	Not exposed to antidepressants (41)	Child Behavioral Checklist for ages 1.5–5 years, filled by parents	Maternal psychopathology, maternal education, gender, gestational age, birth weight, intra-uterine growth restriction, asphyxia (defined as Apgar score at 5 min below 5), maternal smoking and exposure to alcohol during pregnancy.	No significant association between SSRI exposed and offspring internalizing behavior	Prospective longitudinal cohort study (+) Small group sizes (−) Severity of maternal depression/anxiety assessed by self-report (−) Original study plan changed during recruitment (−)
Lupattelli et al., 2018/Norway Norwegian mother and child cohort study (MoBa) Pregnant women recruited to study 1999–2008 (n = 103631) Prospective population-based cohort	5 years 4128	Exposed to SSRI (605)	Unexposed to SSRI (7640)	Child Behavior Checklist, Emotionality, Activity, and Shyness Temperament Questionnaire	Maternal body mass index, parity, education and income, marital status, folic acid use, smoking and alcohol use in pregnancy, illicit substance use, and paternal education, co-medication in pregnancy with analgesics, anxiolytics and sedatives, antipsychotics, and non-SSRI antidepressants, severity of maternal depressive and anxiety symptoms in pregnancy, lifetime history of major depression, child sex, breastfeeding, maternal postnatal mental health	Children exposed to SSRIs in late pregnancy had an increased risk of anxious/depressed behaviors by age 5 years compared with unexposed children (aβ 0.50; 95% CI 0.04–0.96). No association for children exposed to SSRI in mid gestation and anxious/depressed behaviors by age 5 years.	Measured maternal SSRI use during two time points (+) Self-reported maternal depression/anxiety (−) Outcome measures of child development parent-reported (−) Low response rate in MoBa study (41%) (−) Not enough comparison group, important neonatal factors were not considered, follow up time was short (depression). (−) Self-reported use of antidepressants, SSRI.
Grzeskowiak et al., 2016/Denmark Pregnant women recruited to study 1996–2002 (n = 82687) Danish National birth Cohort study (DNBC)	7 years 49178	Antidepressants use (210)	Untreated depression (231) Unexposed (48,737)	Strengths and Difficulties Questionnaire-parental report	Child sex, maternal age, parity, smoking status, alcohol use, and socio-economic status and maternal mood (short version of the Symptom Checklist (SCL–8d))	Antidepressant use associated with internalizing problems (aRR 1.68; 95% CI 1.18–2.38) but after adjusting for maternal mood attenuated the association (aRR 1.20; 95% CI 0.85–1.70)	Prospectively obtained information, women unaware of the study hypothesis at the time of children’s assessment (+) Attempts made to control for maternal underlying illness (+) Due to small sample size, inability to control for several important confounders (−) Parent-reported SDQ score a gross measurement for emotional and behavioral problems, and a potential source of reporting bias (−) Self-reported depression (−) Self-reported use of antidepressants.
Hermansen et al., 2016/Norway MoBa Pregnant women recruited to study 1999–2008 (n = 95200) Prospective population-based cohort	5–6 years 103	SSRI exposed (28)	Depression exposed (Lifetime History of Depression scale Beck Depression Inventory II) (42) Non exposed controls (33)	Child Behavior Checklist Wechsler Preschool and Primary Scale of Intelligence-Revised Neuropsychological Assessment II Attentional Network Task	None.	-Compared to controls, children in the SSRI-exposed group showed significantly more internalizing behavior, t (1,55) = − 2.41, p = 0.018, η^2^ = 0.10) -No difference between the comparison group and depression exposed for internalizing behaviors.	Small sample size (−) Self-report maternal depression (−) High drop out during the follow up (−) No information whether the medication was used prior to, during, or after pregnancy (−) No information on dosages of exposure (−) Self-reported use of SSRI.
Brandlistuen et al., 2015/Norway Pregnant women recruited to study 1999–2010 (n = 108 841) Sibling design (n = 29 762) MoBa	18 and 36 months 20180 siblings at 18 months and 14435 siblings at 36 months	At 18 months: Use of antidepressants (141) At 36 months: Use of antidepressants (112)	At 18 months: no use of antidepressants (20039) At 36 months: no use of antidepressants (14323)	Child Behavior Checklist/2–3	Parity, maternal symptoms of depression during pregnancy (Hopkins Symptom Checklist SCL-5 at week 17), lifetime depression, symptoms of post-partum depression, smoking during pregnancy, alcohol use during pregnancy and co-medication.	At 18 months: In sibling-matched design, prenatal exposure to antidepressants was not associated with internalizing problems. At 36 months: In sibling-matched design, prenatal exposure to antidepressants was associated with increased levels of internalizing behavior problems in the adjusted analyses at 36 months for the subdomain of anxiety (aβ 0.64; 95% CI 0.26, 1.02)	Sibling design adjusting for shared genetic and familial confounding (+) Small sample size prevented stratified analyses (−) No information on dosages of antidepressants use (−) Self-reported maternal depression (−) Low participation rate (40.6%) (−) Self-reported use of antidepressants.
Hanley et al., 2015 Canada Pregnant women recruited from health clinics in Vancouver (191) Longitudinal cohort study	3 and 6 years 110	SRI exposed (44)	SRI non exposed (66)	Child Behavior Checklist, MacArthur Health and Behavior Questionnaire	Maternal depression, sex of the child, maternal drinking during pregnancy, mode of delivery (cesarean section), and prenatal use of other psychotropic medications, and maternal education.	SRI exposure was associated with significantly higher scores on the internalizing behavior (aβ 0.38; 95%CI 0.01, 0.79)) and anxious behaviors subscales (aβ 0.49; 95% CI 0.04, 0.94).	Small sample size (−) Behavioral outcomes based on maternal report (−) Unable to examine timing or duration of SRI exposure on behavioral outcomes (−) Not controlled for neonatal factors (−)
Nulman et al., 2015 Canada Pregnant women from 2005 to 2008 in Motherisk database (225) Prospective cohort	3–6 years, 11 months 45 sibling pairs	Exposed siblings to SRI (45)	Unexposed siblings to SRI (45)	Child Behavior Checklist and Conners’ Parent Rating Scale Wechsler Preschool and Primary Scale of Intelligence–Third Edition	Child’s age, birth order, and severity of maternal depression during pregnancy and after delivery.	Prenatal exposure to SRIs did not predict offspring behavioral outcomes.	Small sample size (−) Mother-reported child behavior questionnaires (−) Maternal self-report on severity of depression (−) Not controlled for neonatal factors (−) Potential recall bias (−) Limited comparison group (−)
El Marroun et al., 2014 Netherlands Pregnant mothers delivering between April 2002-January 2006 (8880) Population-based cohort (Generation R)	1.5,3 and 6 years 5976	Exposed to SSRIs (69)	Exposed to depression (376) Unexposed (5531)	Child Behavior Checklist	Maternal age at intake, gender of the child, maternal education, ethnicity, maternal smoking habits, and gestational age at birth, maternal depressive symptoms at 3 years.	Prenatal SSRI exposure was not associated with child affective problems (OR 1.37; 95% CI 0.87–2.16, P = 0.17). Children exposed to maternal depressive symptoms (without SSRIs) were more likely to have affective problems (OR 1.44; 95% CI 1.15–1.81, P = 0.001)	Information on child problems at multiple time points (+) Small sample size (−) Parent-rated child problems (−) Self-report maternal depression (−) Not controlled for neonatal factors (−) Self-reported use of SSRI and from prescription records.
Pederson et al., 2013 Denmark Pregnant women in Danish birth Cohort (82687) DNBC	4 or 5 years 948	Antidepressants use (127)	Untreated depression (98) Unexposed (723)	Strengths and Difficulties Questionnaire	Maternal age, smoking, alcohol, combined social class, and gender	No significant associations between prenatal antidepressant exposure and behavioral problems.	Self-reported maternal depression (−) Information on behavioral problems was collected prospectively, and the women were unaware of the hypotheses of this study (−) Parent-report child behavioral problems (−) Not controlled for neonatal factors (−) High loss to follow up (−) Risk of type II error due to limited power (−) Self-reported antidepressant exposure.
Nulman et al., 2012 Canada Prospectively collected data in Motherisk program 2001–2006 (608)	3–6 years, 11 months 238	SSRIs during pregnancy (62)	Venlafaxine during pregnancy (62) discontinued pharmacotherapy before conception (54) nondepressed, healthy pregnant women (62)	Child Behavior Checklist and Conners’ Parent Rating Scale Wechsler Preschool and Primary Scale of Intelligence-Third Edition	Antidepressant dose, duration of antidepressant treatment during pregnancy (weeks), severity of depression during pregnancy and at the time of child testing, maternal IQ, and the child’s age and sex.	Dose and duration of antidepressant treatment during pregnancy did not predict child’s internalizing problems.	Small sample size (−) Maternal selfreport of depression in pregnancy and child behavior (−) Children were assessed at one time point (−) Not controlled for neonatal factors (−)
Oberlander et al., 2010 Canada Pregnant mothers referred by physician or self from health clinics (98) Prospective longitudinal study	3 months and 3 years 75	SSRI exposed (33)	Non-SSRI exposed (42)	Child Behavior Checklist	Prenatal and postnatal maternal mood and 5-minute Apgar score	In adjusted model, higher levels of internalizing symptoms were predicted by higher levels of maternal depression symptoms 3 years post-partum (F=5.816, P = 0.02, n^2^ =0.08) but not by prenatal SSRI exposure (F=3.396, P = 0.07, n^2^ =0.05).	Small sample size (−) Mother-rated child behavioral outcomes (−) Limited comparison groups (−) Cohort was a convenience sample, subject to unmeasured confounding including several maternal characteristics (−)
Misri et al., 2006 Canada Pregnant women exposed to SSRI (52) recruited between 1997 and 1999 from British Columbia Women’s Hospital in Vancouver and non-exposed group (23) separately recruited after delivery from pediatricians’ office (75) Prospective cohort	4–5 years 36	SSRI exposed (22)	Non depressed and non-medicated group (14)	Child Behavior Checklist and Child-Teacher Report Form	Maternal depression and anxiety Hamilton Rating Scale for Depression Hamilton Anxiety Rating Scale	-No statistically significant differences in parent or caregiver ratings of internalizing behaviors. -Parental—but not teacher—reports of total child internalizing behaviors associated with maternal symptoms of depression (F=5.43, df=1, 36, p < 0.05) and anxiety (F=6.88, df=1, 36, p < 0.05).	Small sample size (−) Limited comparison groups- did not compare depressed mothers (−)

AD antidepressants; SSRI selective serotonin reuptake inhibitors; SRI serotonin reuptake inhibitor antidepressants; HR hazards ratio; OR odds ratio; RR risk ratio; ICD international classification of diseases

**Table 2:** Description of data sources used in the study.

Organization	Data sources	Data availability (years)	Data retrieved	Data collected for subjects	Data collected years
National Institute for Health and Welfare (THL)	Finnish Medical Birth Register	1987-	Maternal demographic characteristics, medical history reproductive history, health-related behaviors, diagnoses during pregnancy and delivery, and neonatal outcome data up to seven days’ age	Mothers, infants	1996–2018
	Register of Congenital Malformations	1963-	Data on congenital anomalies including live births, stillbirths and fetuses from pregnancy terminations due to severe fetal anomaly, all with at least one detected major congenital anomaly including major structural anomalies, chromosomal defects and congenital hypothyroidism, classified and coded according to the 9th version of the ICD classification.	Infants	1996–2018
	Hospital Discharge Register	1967-	Data on admission and discharge dates as well as primary and secondary diagnoses (two for inpatient care and 20 for outpatient care) at discharge. The register covers all hospital inpatient episodes in public and private institutions and outpatient hospital visits in public hospitals. The diagnoses are coded using ICD-8 (1969–1986), ICD-9 (1987–1995), and ICD-10 since 1996. Data on hospital discharges with ID numbers are available from 1969, and data on all contacts in outpatient clinics are available from 1998 until the present.	Mothers, fathers, children	1969–2018
	Register of primary healthcare visits	2011-	Data on outpatient primary health care, reason for visit, procedures, and follow-up care.	Mothers, fathers, children	2011–2018
Social Insurance Institution in Finland (KELA)	Drug Reimbursement Register	1993-	Date of drug purchase, the International Anatomic-Therapeutic-Chemical (ATC) classification code indicating the generic name of the drug and the dose prescribed.	Mothers, fathers, children (if applicable)	1993–2018
	Special reimbursement register	1964-	Chronic illnesses requiring continuous drug treatment, with additional information on possible special reimbursement status including indication for treatment.	Mothers, fathers, children (if applicable)	1964–2018
Digital and population data services agency	Population Information System	1971-	Basic information, including municipality of residence, marital status, marriages and divorces, and deaths of all Finnish citizens and other citizens residing permanently in Finland	Mothers, fathers, children	1996–2020
Statistics Finland	Education Register	1970-	Completed education. Available annually or at the end of follow-up.	Mothers, fathers, children	1996–2018

**Table 3:** Covariates to be tested in the study.

Covariates	Categorization	Information retrieved
**Maternal factors**		
Maternal age	Continuous (years) ≤ 19 years, 20–29 years (ref), 30–39 years, ≥ 40 years	MBR, Population register
Place of residence	Urban, semiurban, rural	MBR
Marital status	Married or in a relationship, divorced vs. unmarried)	MBR
Maternal SES	Upper white-collar worker, lower white-collar worker, blue-collar worker, other, unknown	MBR
Maternal education	mother’s highest level of education (child’s birth year)	Education register
Maternal unemployment status	Employed, unemployed	Finnish Pension center
Maternal death	Yes, no	Population register
Maternal parity	No previous births; 1 or more previous births	MBR
Maternal smoking	Yes, no	MBR
Artificial reproductive technology (ART) in the present pregnancy	Yes, no	MBR
Pre-eclampsia	Yes, no	MBR
Hypertension	Yes, no	MBR
Maternal pre-pregnancy BMI (available 2004 onwards)	< 18.5, 18.5–24.9 (ref), 25–29.9, ≥ 30	MBR
Pre-pregnancy diabetes (ICD-10 E10-E14 recorded before LMP; ICD-9 250; ICD-8 250	Yes, no	MBR, HDR
Number of hospitalizations (other than psychiatric)	1, 2, ≥ 3	HDR
Number of outpatient contacts (other than psychiatric)	1, 2, ≥ 3	Primary health care registers
Maternal purchase of known teratogens, any time during pregnancy or 1 month before pregnancy	Yes, no	Social Insurance institution, DPR
Maternal purchase of anxiolytics and/or sedatives, any time during pregnancy or 1 month before pregnancy	Yes, no	Social Insurance institution, DPR
Maternal purchase of antiepileptic drugs, any time during pregnancy or 1 month before pregnancy (yes, no)	Yes, no	Social Insurance institution, DPR
Maternal psychiatric diagnoses excluding depression or depression-related disorders (ICD-10 F20-F48; ICD-9 295–298).	Yes, no	HDR
Bleeding during or after delivery (during ICD-10 O67 (ICD-10 O67.0; 67.8; 67.9; after ICD-10 O72 (ICD-10 O72.0–72.3)	Yes, no	MBR
**Paternal factors**		
Paternal age	Continuous (years) ≤ 19 years, 20–29 years (ref), 30–39 years, ≥ 40 years	Population register
Paternal SES	Upper white-collar worker, lower white-collar worker, blue-collar worker, other, unknown	Statistics Finland
Paternal education	Father’s highest level of education (child’s birth year)	Education register
Paternal unemployment status	Employed, unemployed	Finnish Pensions Center
Paternal death	Yes, no	Population register
Paternal psychopathology	Yes, no	HDR
Paternal SSRI use	Yes, no	DRR
**Neonatal factors**		
Child’s sex	Boy, girl	MBR
Birth year	Years	MBR
Gestational age	Extremely preterm, preterm, late preterm, term, post term	MBR
Birth weight	Very low birth weight, low birth weight, normal	MBR
Birth weight for gestational age	Small for gestational age, appropriate for gestational age, large for gestational age	MBR
Apgar score at 5 min	0–6, 7–10, missing	MBR
Mode of delivery	Vaginal, instrumental, cesarean	MBR
Neonatal breathing problems ICD-10 P20-P28	Yes, no	MBR
Monitoring at NICU	Yes, no	MBR
PPHN ICD-10 P29.31	Yes, no	MBR, HDR
Hospital stay at 7 days of age	Yes, no	MBR
Major congenital anomalies	Yes, no	Malformation register
Any first-degree history of psychiatric disorder	Yes, no	HDR
